# 400. Predictors of Post-COVID Conditions after SARS-CoV-2 Infection in Adults Enrolled in a Multistate Household Transmission Study

**DOI:** 10.1093/ofid/ofad500.470

**Published:** 2023-11-27

**Authors:** Michelle Floris-Moore, Melissa Stockwell, Carlos G Grijalva, H Keipp Talbot, Alexandra Mellis, Sarah E Smith-Jeffcoat, Melissa A Rolfes, Sharon Saydah, Jessica E Biddle, Jessica T Lin, Jonathan Schmitz, James Chappell, Kimberly W Hart, Son H McLaren, Ellen Sano, Lisa Saiman, Yvonne A Maldonado, Katherine Ellingson, Karen Lutrick, Huong McLean, Edward Belongia, Zoe Martin del campo, Ayla Bullock, Suchitra Rao, Edwin J Asturias, Natalie M Bowman

**Affiliations:** University of North Carolina at Chapel Hill, Chapel Hill, North Carolina; Columbia University Irving Medical Center, New York City, New York; Vanderbilt University Medical Center, Nashville, Tennessee; Vanderbilt University Medical Center, Nashville, Tennessee; Centers for Disease Control and Prevention, Atlanta, GA; Centers for Disease Control and Prevention, Atlanta, GA; Centers for Disease Control and Prevention, Atlanta, GA; Centers for Disease Control and Prevention, Atlanta, GA; Centers for Disease Control and Prevention, Atlanta, GA; University of North Carolina at Chapel Hill, Chapel Hill, North Carolina; Vanderbilt University Medical Center, Nashville, Tennessee; Vanderbilt University Medical Center, Nashville, Tennessee; Vanderbilt University Medical Center, Nashville, Tennessee; Columbia University Irving Medical Center, New York City, New York; Columbia University Irving Medical Center, New York City, New York; Columbia University Irving Medical Center, New York City, New York; Stanford University, Stanford, California; University of Arizona, Tucson, Arizona; University of Arizona College of Medicine, Tucson, Arizona; Marshfield Clinic Research Institute, Marshfield, WI; Marshfield Clinic, Marshfield, Wisconsin; University of North Carolina Gillings School of Global Public Health, Chapel Hill, North Carolina; University of North Carolina, Chapel Hill, North Carolina; University of Colorado School of Medicine, Aurora, Colorado; University of Colorado School of Medicine, Aurora, Colorado; University of North Carolina, Chapel Hill, North Carolina

## Abstract

**Background:**

Severe COVID-19 predicts increased risk of Post-COVID conditions (PCC). However, the impact of mild COVID-19 in non-hospitalized patients on development of PCC is less clear.

**Methods:**

We recruited individuals with mild SARS-CoV-2 (SCV2) infection from 7 US sites into a household transmission study, Sep. 2021–Dec. 2022. Index cases and their household contacts (HHC) were enrolled ≤6 days after the index case tested positive for SCV2. At baseline, participants provided sociodemographic, clinical and vaccine history, and dried blood spot for antibody detection. Participants completed daily symptom and medication diaries and collected nasal swabs for quantitative PCR (qPCR) for 10 days as well as a 90-day survey including the PROMIS® Global Health measure of physical, mental, and social health. We defined PCC as presence of ≥1 symptom (Table 1) most or almost all of the time 90 days post enrollment (Fig. 1). We calculated the proportion of PCC among adults with SCV2 infection and evaluated factors associated with PCC using Chi-squared, Student’s t-test, or binary logistic regression, as applicable

**Table 1:**
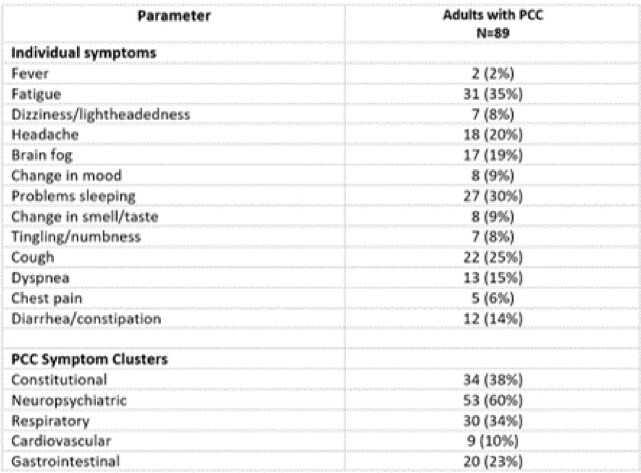
Individual symptoms and symptom clusters occurring most of the time or almost all the time at 90-day follow-up among adults who had Post-COVID (PCC) conditions

At 90-days after enrollment, participants were asked how often they experience a list of 17 symptoms with response options of never, sometimes, most of the time, almost all the time. Those who responded most or almost all the time to at least one of the listed symptoms and tested positive for SARS-CoV-2 during enrollment or daily swabbing were defined as having PCC. Constitutional symptoms include fever, fatigue, and dizziness/lightheadedness; Neuropsychiatric symptoms include brain fog, change in mood, problems sleeping, tingling/numbness, and dizziness/lightheadedness; Respiratory symptoms include cough and dyspnea; Cardiovascular symptoms include chest pain and dizziness/lightheadedness; Gastrointestinal symptoms include change in taste/smell, vomiting, constipation, and diarrhea.

**Figure 1.**
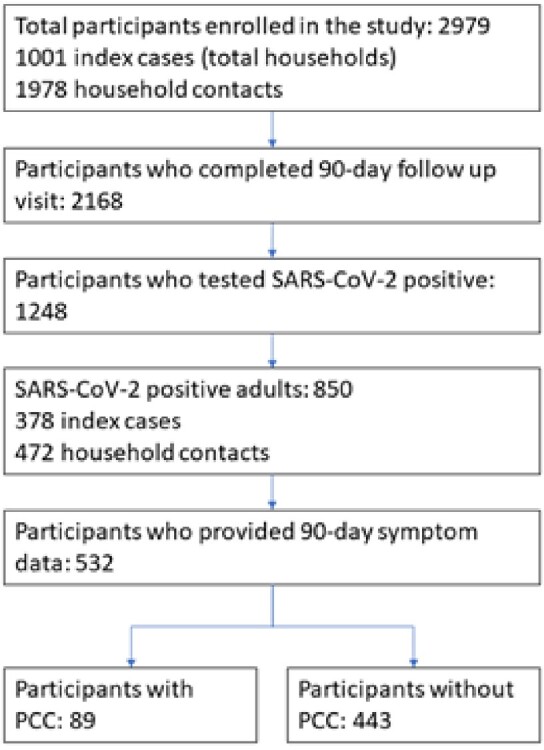
Flow chart of participants who tested positive for SARS-CoV-2 and completed a 90-day survey for Post-COVID conditions (PCC)

**Results:**

Among 532 adults with SCV2 who completed a 90-day survey, 326 (61.3%) were female, mean age was 47.4 years (SD 16.8), and 89 (17%) met PCC definition. Among people with PCC, the most common symptoms were neuropsychiatric (60%), fatigue (35%), respiratory (34%), and sleep problems (30%; Table 1); 41% reported ≥ 2 symptoms and 23% reported ≥ 3. HHC had lower odds of PCC compared to index cases (OR 0.6, 95% CI 0.4, 0.9), college graduates had higher odds of PCC compared to non-graduates (OR 1.7, 95% CI 1.0, 2.9) and participants with comorbidities had twice the odds of PCC compared to those without (95% CI 1.2, 3.4; Table 2). There were no significant associations between viral load, antibodies, or treatment during acute illness and PCC (Table 3). PCC was associated with higher odds of reporting poor/fair quality of life (OR 7.8, 95%CI 2.9, 21.3), physical health (OR 8.2, 95%CI 4.2, 16.3), mental health (OR 4.9, 95%CI 2.7, 8.9), and social satisfaction (OR 4.9, 95%CI 2.5, 9.6).

**Table 2:**
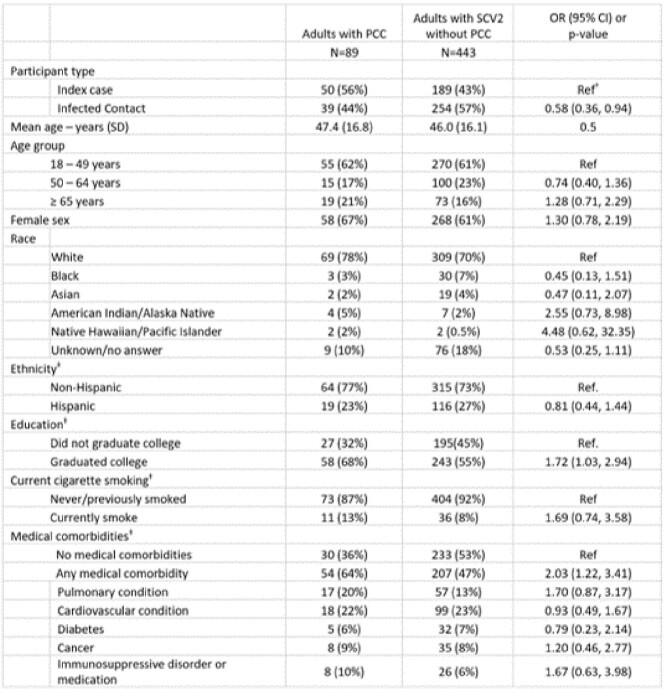
Characteristics of adult participants who had SARS-CoV-2 infection and who did and did not develop Post-COVID conditions (PCC)* at 90-day follow up SCV2: SARS-CoV-2; OR: Odds ratio; CI: Confidence interval * PCC defined as presence of ≥ 1 of the following symptoms most or all of the time, 90 days after enrollment: fever, fatigue or post-exertional malaise, weight loss, mood changes, headache, tingling/numbness, change in ability to smell/taste, cough, shortness of breath, chest pain/palpitations, lightheadedness/dizziness, syncope, change in appetite, nausea, vomiting, diarrhea, constipation, or sleep disturbance. † Ref: Reference category ‡ Data on ethnicity available for 514 participants (83 with PCC and 431 without PCC). Data on level of education available for 523 participants (85 with PCC and 438 without PCC). Data on current cigarette smoking and medical comorbidities available for 524 participants (84 with PCC and 440 without PCC).

**Table 3:**
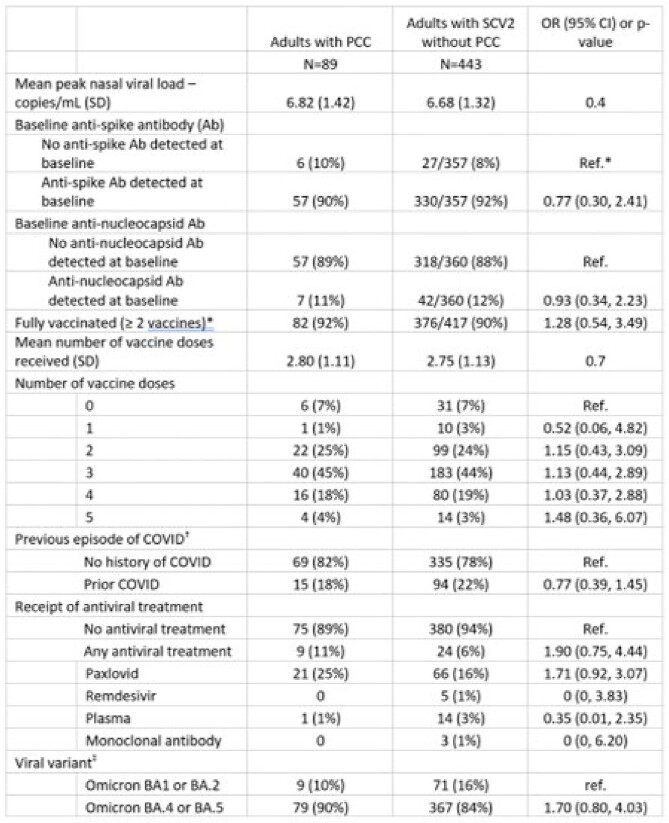
Virologic, immunologic and treatment characteristics of adult participants who had SARS-CoV-2 infection and who did and did not develop Post-COVID conditions (PCC)* at 90-day follow up SCV2: SARS-CoV-2; OR: Odds ratio; CI: Confidence interval, Ref.: Reference category *Fully vaccinated defined as having received at least two doses of a COVID vaccine (inclusive of all mRNA, vector, or nanoparticle COVID vaccines) † History of COVID prior to the SARS-CoV-2 infection that qualified the participant for study enrollment. ‡ Data on viral variant available for 526 participants (88 with PCC and 438 without PCC).

**Conclusion:**

In this household transmission study, 17% of ambulatory adults with COVID-19 reported PCC symptoms 90 days after acute infection. Index cases, people with comorbidities, and college graduates had higher odds of PCC.

**Disclosures:**

**Michelle Floris-Moore, MD, MS**, Viiv Healthcare: Advisor/Consultant **Carlos G. Grijalva, MD, MPH**, AHRQ: Grant/Research Support|CDC: Grant/Research Support|FDA: Grant/Research Support|Merck: Advisor/Consultant|NIH: Grant/Research Support|Syneos Health: Grant/Research Support **Lisa Saiman, MD MPH**, Merck & Co., Inc,: Grant/Research Support|Merck & Co., Inc,: Member, DSMB|Pfizer, Inc: Member, DSMB **Yvonne A. Maldonado, MD**, Pfizer: Grant/Research Support|Pfizer: Site Investigator, DSMB member **Huong McLean, PhD, MPH**, Seqirus: Grant/Research Support **Suchitra Rao, MBBS, MSCS**, Sequiris: Advisor/Consultant **Edwin J. Asturias, MD**, Hillevax: Advisor/Consultant|Moderna: Advisor/Consultant|Pfizer: Grant/Research Support

